# The volume of infusion fluids correlates with treatment outcomes in critically ill trauma patients

**DOI:** 10.3389/fmed.2022.1040098

**Published:** 2023-01-12

**Authors:** Anna Wrzosek, Tomasz Drygalski, Jarosław Garlicki, Jarosław Woroń, Wojciech Szpunar, Maciej Polak, Jakub Droś, Jerzy Wordliczek, Renata Zajączkowska

**Affiliations:** ^1^Department of Interdisciplinary Intensive Care, Jagiellonian University Medical College, Kraków, Poland; ^2^Department of Anaesthesiology and Intensive Therapy, University Hospital, Kraków, Poland; ^3^Department of Anaesthesiology and Intensive Therapy, Jagiellonian University Medical College, Kraków, Poland; ^4^Department of Clinical Pharmacology, Medical College, Jagiellonian University, Kraków, Poland; ^5^Department of Epidemiology and Population Studies, Jagiellonian University Medical College, Kraków, Poland; ^6^Doctoral School in Medical and Health Sciences, Jagiellonian University Medical College, Kraków, Poland

**Keywords:** fluid overload, fluid resuscitation, critical illness, trauma, injury, pulmonary edema, cardiac failure

## Abstract

**Background:**

Appropriate fluid management is essential in the treatment of critically ill trauma patients. Both insufficient and excessive fluid volume can be associated with worse outcomes. Intensive fluid resuscitation is a crucial element of early resuscitation in trauma; however, excessive fluid infusion may lead to fluid accumulation and consequent complications such as pulmonary edema, cardiac failure, impaired bowel function, and delayed wound healing. The aim of this study was to examine the volumes of fluids infused in critically ill trauma patients during the first hours and days of treatment and their relationship to survival and outcomes.

**Methods:**

We retrospectively screened records of all consecutive patients admitted to the intensive care unit (ICU) from the beginning of 2019 to the end of 2020. All adults who were admitted to ICU after trauma and were hospitalized for a minimum of 2 days were included in the study. We used multivariate regression analysis models to assess a relationship between volume of infused fluid or fluid balance, age, ISS or APACHE II score, and mortality. We also compared volumes of fluids in survivors and non-survivors including additional analyses in subgroups depending on disease severity (ISS score, APACHE II score), blood loss, and age.

**Results:**

A total of 52 patients met the inclusion criteria for the study. The volume of infused fluids and fluid balance were positively correlated with mortality, complication rate, time on mechanical ventilation, length of stay in the ICU, INR, and APTT. Fluid volumes were significantly higher in non-survivors than in survivors at the end of the second day of ICU stay (2.77 vs. 2.14 ml/kg/h) and non-survivors had a highly positive fluid balance (6.21 compared with 2.48 L in survivors).

**Conclusion:**

In critically ill trauma patients, worse outcomes were associated with higher volumes of infusion fluids and a more positive fluid balance. Although fluid resuscitation is lifesaving, especially in the first hours after trauma, fluid infusion should be limited to a necessary minimum to avoid fluid overload and its negative consequences.

## 1. Introduction

Appropriate fluid management is essential in the treatment of critically ill trauma patients. Recent studies have highlighted that both insufficient and excessive volumes are associated with increased mortality (1–3). Fluid resuscitation in trauma is crucial in the early phase of treatment when it is necessary to restore adequate intravascular volume in hemorrhagic shock. Positive fluid balance is expected and beneficial to patients at this stage and may help restore adequate blood pressure and tissue perfusion, but the volumes used usually exceed the amount needed to restore blood loss (4). However, excessive fluid administration in consequence leads to fluid accumulation, which is associated with several serious complications including pulmonary edema, cardiac failure, delayed wound healing, tissue breakdown and impaired bowel function (1, 5). It can worsen metabolic acidosis (6), aggravate hypothermia, and lead to coagulopathy, very often seen in bleeding patients (trauma-induced coagulopathy; TIC), which can further increase bleeding (7, 8). Nethertheless, even though all experts agree that both fluid excess and fluid limitation are harmful to patients, the “right amount” still remains an open question.

Many study authors recently suggested that restrictive fluid therapy (RFT) may be better for patients (9–12). There are publications that compare restrictive and liberal fluid therapy in perioperative setting in non-trauma surgery. Most of them show superiority of restrictive approach, while only few show an increased complication rate in the restrictive group (13, 14). However, whether RFT is also beneficial in trauma patients has not been clearly established in clinical studies, and some of the results are contradictory (15–17). The aim of our study was to examine volumes of fluids infused in critically ill trauma patients during the first hours and days of treatment and their relationship to survival and outcomes.

## 2. Materials and methods

After obtaining a consent of the Jagiellonian University Ethics Committee, we screened records of all consecutive patients admitted to intensive care unit (ICU) from the beginning of 2019 to the end of 2020. Adults who were admitted to ICU after trauma were included in the study. The exclusion criterion was death before the end of day 2 after admission to the ICU. We retrospectively extracted clinical and preclinical data from electronic and traditional (paper) records of the patients. Data included pre-hospital medical records, emergency department (ED), operating theater (OT), and ICU records. All data was collected and analyzed anonymously.

We extracted the following patients’ data: age, weight, Injury Severity Score (ISS), APACHE II score, Glasgow Coma Scale (GCS), Revised Trauma Score (RTS) volume of fluids infused, volume of blood products transfused, fluid loss, blood loss, fluid balance, mortality, ventilator free days, total number of complications, time until ready for discharge from ICU, time of continuous renal replacement therapy (CRRT), lactate level, hematocrit level, platelet count, activated partial thromboplastin time (APTT), international normalized ratio (INR), pH, base excess. ISS, APACHE II, and RTS scores were obtained with the use of online calculators (18, 19). The APACHE II score was calculated at admission to the ICU unit, GCS, and RTS were obtained at the site of injury by the emergency team. All fluid data was obtained until the end of day 2 and included pre-hospital period, ED, surgery, and ICU stay. All laboratory results were obtained on days 0 and 2. Volume of infused fluids (fluid volume) was defined as sum of fluids infused intravenously including drug diluents, but not including blood products until the end of the second day of stay in the ICU (including pre-hospital, ED, and OT time). Volume of blood products transfused included red blood cell concentrate, fresh frozen plasma, and platelets. Fluid loss included diuresis, drainage loss, diarrhea, residual gastrointestinal volume, insensible perspiration. Insensible perspiration was estimated to be 7 ml per kg of actual body weight, and an additional 500 ml was added in the event of an increase in temperature to more than 38 degrees Celsius. Fluid balance was calculated by subtracting fluid loss and blood loss from the sum of fluid volume and blood products and fluids administered per gastrointestinal tract up to the end of the second day of stay in ICU (including pre-hospital, ED, and OT time). Mortality was defined as number of deaths until day 28 after trauma, including deaths in ICU, in the hospital, and after discharge. Ventilator free days were defined as number of days without mechanical ventilation until day 28 after trauma. The higher number of ventilator free days indicates shorter time on mechanical ventilation. Time until ready for discharge was analyzed until day 28. It was defined as the time of stay in the ICU until the patient was directed for discharge by the attending physician. It did not include the waiting time for a place for the patient on another hospital ward due to organizational aspects of care. Time of CRRT was defined as a total time when patients were on CRRT expressed in hours. In case of missing data, last observation carried forward imputation method was used when possible.

The study was carried out according to the STROBE (Strengthening the Reporting of Observational Studies in Epidemiology) guidelines (20).

## 3. Statistical analysis

The distribution of categorical variables was presented as numbers and percentages, numerical as means with standard deviations for normally distributed continuous variables, and as medians with interquartile ranges for continuous variables with a skewed distribution. The multivariable logistic regression models were used to assess a relationship between fluid volume (ml/kg/h) or fluid balance (liters), age, ISS or APACHE II score, and mortality. The results of the logistic regression model were presented as odd ratios (ORs) and 95% confidence intervals (CI). The Poisson regression model was used to assess the relationships between cumulative fluid volume or fluid balance and ventilator free days, total number of complications, time until ready for discharge from ICU, time of CRRT, volume of blood products transfused, APTT and INR. The results of Poisson regression were presented as incidence rate ratio (IRR) and 95% CI. Spearman correlation was used asses the relationships between numerical variables. For the calculation purposed in the regression and correlation analysis, the time until ready for discharge was established at a value of 28 both for patients who were hospitalized in the ICU for more than 28 days and for patients who died before day 28, to reflect the real situation, as these patients have never left the ICU. The Kolmogorov-Smirnov test was used to verify the normality of the data distribution. The student’s *t*-test, Mann–Whitney and chi-square test was used to compare groups of survivors and non-survivors. The calculation of the minimum sample size was based on patients’ data of the patients in the study by van Mourik et al. (21), where two and a half times the difference in fluid balance was observed in survivors and non-survivors. We considered such differences clinically relevant. The sample size calculation suggested a minimum of 44 patients (23 in each group) to confirm or reject the hypothesis for the primary endpoint (mortality) with 80% power and 95% significance level. All analyzes were performed in SPSS version 24.0 for Windows (SPSS, Chicago, IL, USA). Statistical significance was defined as *p* ≤ 0.05.

## 4. Results

**FIGURE 1:**
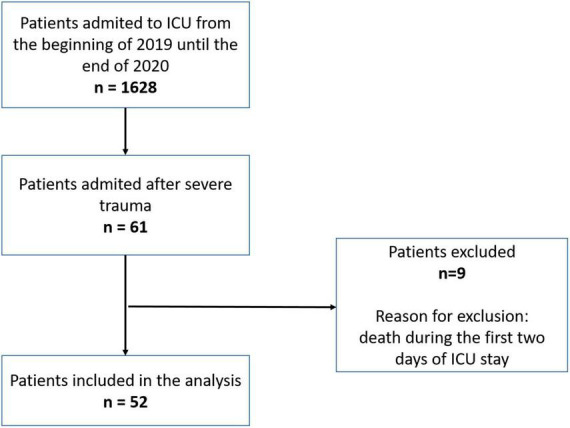
Patient flow diagram.

A total of 1,628 patients were admitted to ICU from the beginning of 2019 until the end of 2020. Of this group, 61 patients were admitted after severe trauma. Nine patients died before the end of the second day of stay in the ICU and 52 patients were included in the study. Patient flow diagram is presented in Figure 1. Summary of clinical data for the patients is presented in Table 1. The mean age of the included patients was 44 years. Most of the patients were men (77%). The mean ISS was 62.2. Most of the patients had chest injuries (94%), and 71% had head injuries. Abdominal, pelvic, and limb injuries were observed in 54, 31, and 73% of the patients 75% of patients were operated. The 28-day mortality was 23% (12 patients), and the mean time to death was 9.41 days. The mean volume of infused fluids, including pre-hospital setting, surgery time, and the first 2 days of stay in the ICU, was 15.6 L total and 2.28 ml/kg body weight/hour. The median fluid balance at the end of stay in the second day of ICU was + 2704 ml. Most of the infused fluids were balanced crystalloids. The median volume of blood products transfused was 0.78 L.

**TABLE 1:** Summary of clinical data for all patients.

Variable	Outcome
Age [mean (SD)]	44.1 (18.4) years
Weight [mean, (SD)]	78.5 (11.3) kg
Males [*n*, (%)]	40 (77%)
ISS [mean, (SD)]	62.2 (9.3)
APACHE [mean, (SD)]	22.79 (5.89)
GCS [mean, (SD)]	7.35 (2.9)
RTS [mean, (SD)]	5.01 (1.09)
Type of injury [*n*, (%)]	Head injury–37 (71%)
	Chest injury–49 (94%)
	Abdominal injury–28 (54%)
	Pelvic injury–16 (31%)
	Limb injury–38 (73%)
Patients who had surgery	39 (75%)
Total volume of fluids [mean (SD)]	15 642 ml (5921)
Volume of fluids per kg of body weight per hour [mean (SD)]	2.28 ml/kg/h (0.79)
Fluid balance [median (Q1–Q3)]	+ 2704 ml (690–5856)
Fluid balance per kg of body weight [median (Q1–Q3)]	36.1 ml/kg (8.6–72.97)
Total volume of blood products transfused [median (Q1–Q3)]	779.58 ml (0–20545)
Total volume of blood products transfused per kg body weight [median (Q1–Q3)]	9.0 ml/kg (0–29.0)
Blood loss [median (Q1–Q3)]	230.0 ml (0–862.5)
Blood loss per kg body weight per hour (ml/kg/h) [median (Q1–Q3)]	2.9 ml/kg/h (0–10)
Patients who needed CRRT [*n*, (%)]	21 (49%)
28-day mortality [*n*, (%)]	12 (23%)
28-day ICU mortality [*n*, (%)]	11 (21%)
Days until ready for discharge from ICU [mean (SD)]	11.7 (10.5)
Ventilator free days until day 28 [mean (SD)]	11.7 (10.5)
Patients with complications [*n*, (%)]	44 (85%)
Type of complications [*n*, (%)]	Cardiovascular–11 (21%)
	Pulmonary–27 (52%)
	Sepsis–10 (19%)
	Renal–22 (42%)
	Gastrointestinal–31 (60%)
	Neurological–2 (4%)
	Other–2 (4%)
Lactate level, day 0 [median (Q1–Q3)]	2.35 (1.1–3.8)
Hematocrit level, day 0 [mean (SD)]	32.36% (7.39%)
Platelet count, day 0 [median (Q1–Q3)]	172 (126–191)
APTT level, day 0 [median (Q1–Q3)]	31.6 (27.9–35.5)
INR level, day 0 [median (Q1–Q3)]	1.17 (1.11–1.34)
Base excess, day 0 [median (Q1–Q3)]	−4.4 (−6.25–1.75)
pH, day 0 [mean (SD)]	7.31 (0.07)
Lactate level, day 2 [median (Q1–Q3)]	1.4 (0.9–2.4) mmol/l
Hematocrit level, day 2 [mean (SD)]	28.4% (5.7%)
Platelet count, day 2 [median (Q1–Q3)]	130 (93–170)
APTT level, day 2 [mean (SD)]	33.6 (10.7)
INR level, day 2 [mean (SD)]	1.13 (0.4)
Base excess, day 2 [median (Q1–Q3)]	2.4 (−0.1–4)
pH, day 2 [mean (SD)]	7.41 (0.06)

Values presented as means with SD in brackets or median (first quartile–third quartile). ISS, injury severity score; GCS, Glasgow coma scale; CRRT, continuous renal replacement therapy; ICU, intensive care unit; APTT, activated partial prothrombin time; INR, international normalized ratio.

In a multivariable logistic regression analysis volume of the infused fluids and the fluid balance were positively associated with mortality (OR = 4.10; 95% CI = 1.030–16.317 and OR = 1.236; 95% CI = 1.008–1.515, respectively, Table 2). For other factors analyzed (age, ISS, APACHE II score) the association was not detected. Additionally, volume of fluids was negatively correlated with number of ventilator free days and fluid balance was negatively correlated with ventilator free days, and positively correlated with complications and time until ready for discharge from ICU (Table 3). Furthermore, volume of fluids and/or fluid balance was correlated with volume of blood products transfused, INR, and APTT (Table 3).

**TABLE 2:** Multivariable logistic regression analysis of risk factors associated with mortality in critically ill trauma patients.

Predictor	OR	95% CI	*P*-value
**Model 1 including volume of fluids**			
Volume of fluids	4.100	1.030–16.317	*p* = 0.040*
Age (years)	1.058	0.999–1.120	*p* = 0.053
ISS	0.983	0.904–1.070	*p* = 0.689
APACHE II	1.131	0.940–1.361	*p* = 0.180
**Model 2 including fluid balance**			
Fluid balance (liters)	1.236	1.008–1.515	*p* = 0.037*
Age (years)	1.039	0.989–1.092	*p* = 0.117
ISS	1.004	0.920–1.095	*p* = 0.928
APACHE II	1.171	0.980–1.399	*p* = 0.074

OR, odds ratio; CI, confidence interval; ISS, injury severity score. *Results statistically significant.

**TABLE 3:** Relationship between volume of fluids (ml/kg/h) or fluid balance (liters) and outcomes in critically ill trauma patients.

	Volume of fluids		Fluid balance	
**Outcome**	**IRR (95% CI)**	***P*-value**	**IRR (95% CI)**	***P*-value**
Ventilator free days	0.75 (0.67–0.85)	*P* < 0.001*	0.91 (0.89–0.93)	*p* < 0.001*
Total number of complications	1.05 (0.83–1.31)	*P* = 0.70	1.06 (1.02–1.10)	*p* = 0.004*
Time until ready for discharge from ICU (days)	1.07 (0.99–1.15)	*p* = 0.76	1.03 (1.01–1.05)	*p* < 0.001*
	**R**	***P*-value**	**R**	***P*-value**
Time of CRRT	−0.01	*p* = 0.96	0.24	*p* = 0.09
Volume of blood products transfused (ml/kg)	0.55	*p* < 0.01*	0.70	*p* < 0.001*
APTT (seconds)	0.31	*p* = 0.057	0.53	*p* < 0.001*
INR	0.50	*p* < 0.01*	0.62	*p* < 0.001*

IRR, incidence rate ratio; R, correlation coefficient; ICU, intensive care unit. *Results statistically significant.

**FIGURE 2:**
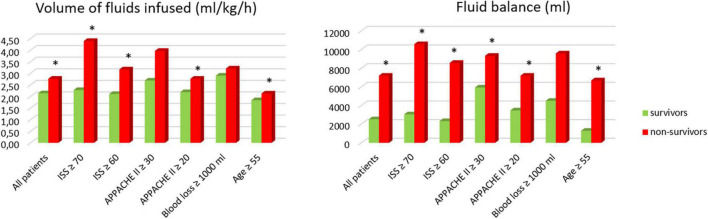
Comparison between survivors and non-survivors. *Results statistically significant.

Comparison between survivors and non-survivors showed that non-survivors received significantly more fluid than survivors: 2.77 (1.03) vs. 2.14 (0.61) ml/kg/h and had significantly more positive fluid balance (6.21 vs. 2.48 L). Analyses in subgroups depending on the severity of the disease (ISS, APACHE II score), blood loss, and age also showed significant differences in majority of subgroups (Figure 2 and Table 4).

**TABLE 4:** Comparison between survivors and non-survivors.

Variable	Survivors	Non-survivors	*P*-value
Age (years) [mean (SD)]	41.9 (17.0)	51.25 (17.03)	*p* = 0.13
Weight (kg) [mean (SD)]	79.3 (11.47)	75.75 (10.74)	*p* = 0.35
ISS [mean (SD)]	61.83 (10.79)	57.25 (14.19)	*p* = 0.24
APACHE II [mean (SD)]	21.43 (5.63)	27.33 (4.50)	*p* = 0.002*
Volume of fluids (ml/kg/h) [mean (SD)]			
All patients	2.14 (0.61)	2.77 (1.03)	*p* = 0.013*
ISS ≥ 70	2.28 (0.40)	4.40 (0.18)	*p* < 0.01*
ISS ≥ 60	2.11 (0.51)	3.17 (1.24)	*p* = 0.01*
APACHE II ≥ 30	2.69 (0.42)	3.97 (0.86)	*p* = 0.05*
APACHE II ≥ 20	2.19 (0.67)	2.77 (1.03)	*p* = 0.05*
Blood loss ≥ 1000 ml	2.90 (0.39)	3.21 (1.58)	*p* = 0.61
Age ≥ 55	1.84 (0.68)	2.13 (0.33)	*p* = 0.28
Fluid balance (ml) [median (Q1–Q3)]			
All patients	2482.5 (654.5–4149.0)	6213.5 (3199.5–12810)	*p* < 0.01*
ISS ≥ 70	3056.22 (1801.96)	10588.50 (5929.09)	*p* < 0.01*
ISS ≥ 60	2560.0 (766.0–3830.0)	8833 (4133–13025.5)	*p* = 0.03*−
APACHE II ≥ 30	6262 (2560–9593)	6930 (4529–14781)	*p* = 0.55
APACHE II ≥ 20	3146 (1665–5465)	6213 (3199–12810)	*p* = 0.04*
Blood loss ≥ 1000 ml	3310 (1665–9593)	8649 (5279–13025)	*p* = 0.07
Age ≥ 55	963 (−1144–2613)	6930 (−105–14350)	*p* = 0.12
Blood loss (ml) [median (Q1–Q3)]	200 (0–725)	420 (0–1195)	*p* = 0.73
Ventilator free days [mean (SD)]	22.4 (11.34)	0 (0)	*p* < 0.01*
Total number of complications [mean (SD)]	2.15 (1.85)	2.75 (1.86)	*p* = 0.33
Time until ready for discharge from ICU [mean (SD)]	18.58 (9.02)	28.00 (0)	*p* < 0.01*
Time to death [mean (SD)]		9.41 (10.78)	

Values presented as means with SD in brackets or median (first quartile–third quartile). ISS, injury severity score; ICU, intensive care unit. *Results statistically significant.

## 5. Discussion

In our study, we demonstrated an association between fluid volumes received by critically ill trauma patients during the first hours and days of treatment and treatment outcomes, including mortality, mechanical ventilation, and length of ICU stay. Additionally, non-survivors received much more fluid than survivors and had substantially higher positive fluid balance. Intensive fluid resuscitation is essential for patients in hemorrhagic shock after trauma, however, it can lead to fluid overload, which in consequence, can worsen the condition and lead to serious complications, including pulmonary edema, cardiac failure, delayed wound healing, tissue breakdown and impaired bowel function. Endothelial glycocalyx shading and increased vascular permeability caused by hemorrhagic shock and systemic inflammatory response syndrome (SIRS) associated with trauma can be further aggravated by aggressive fluid resuscitation (22). Thus, fluid resuscitation should be limited to the minimal necessary volumes and stopped as soon as possible with deresuscitation started immediately after the patient’s condition stabilizes. Vasopressor infusion should be started without unnecessary delay to restore adequate blood pressure and avoid unnecessary fluid transfusion. Blood loss should be restored with blood products as soon as available (2, 23, 24). For other factors included in the models (age, ISS score, and APACHE II score), the analysis did not show a statistically significant association between these variables and mortality, which could have been expected. However, it should be taken into account that this could be caused to some extent by limitations associated with a relatively small simple size in our study and the lack of adequate statistical power in the models. In our study, cumulative infusion volumes were significantly higher in non-survivors (2.77 vs. 2.14 ml/kg/h) and cumulative fluid balance was 2.5 times higher in non-survivors (6.21 vs. 2.48 L). In subgroups divided by the severity of the disease (ISS, APACHE II score), blood loss, and age, cumulative volumes of fluids and fluid balance also differed significantly between survivors and non-survivors. Addressing possible confounding factors is not a direct proof of a causative association between fluid volumes and worse outcomes; however, it suggests that such an implication may be present. The blood loss observed in our study was less than expected. This is probably caused by underestimation, since it is very difficult to accurately estimate real blood loss in trauma victims (25). Our findings remain in correspondence with other studies that show similar results. Hussmann et al. in pre-hospital setting showed that volume of fluids administered in trauma patients is an independent risk factor for mortality (26, 27). Brinck et al. and Piekarski et al. demonstrated that the implementation of blood transfusion and fluid resuscitation protocols leads to decreased mortality, which was accompanied by a decrease in fluid transfusion and an increased ratio of blood products to cristalloids (28, 29). The median cumulative fluid balance in our patients was + 2704 ml and in some patients it was even + 14 781 ml, which shows that the patients received a lot of extra fluid. This surely had to lead to fluid accumulation and consequent complications, such as pulmonary edema, cardiac failure, delayed wound healing, tissue breakdown, and impaired bowel function (5). Furthermore, excessive fluid transfusion leads to hypothermia, acidosis, and coagulopathy. It leads to immunological and inflammatory mediator dysfunction, and depending on the type of fluid infused, these pathological changes may be further aggravated by hypocalcemia and hyperchloremia. Excess fluid accumulation in the abdominal compartment contributes to increased intraabdominal pressure, which in turn deteriorates function of intraabdominal organs, especially encapsulated ones such as the liver and kidneys (30). Balogh et al. demonstrated that supranormal fluid resuscitation (with the aim of a higher oxygen delivery index) in trauma patients was associated with an increased incidence of intraabdominal hypertension, multiple organ failure, and death (31). These findings from clinical trials have additionally been confirmed in mathematical models set to maximize oxygen delivery to tissues. These models showed that the heart oxygen delivery rate was higher at lower fluid infusion rates and that fluid overload had a negative effect. The explanation for these might be the fact that the positive hemodynamic effect did not surpass the negative consequences of hemodilution and decreased hematocrit (32–34). The dysfunctions mentioned above may be responsible for increased morbidity and mortality in trauma patients treated with liberal fluid infusions (8).

Our findings are in correspondence with other studies. For non-emergency surgeries, many authors showed that liberal (excessive) fluid therapy in perioperative setting decreases survival of patients (10, 35, 36). For traumatic injuries, the data is inconsistent and high quality evidence is lacking. Many clinical trials performed in a pre-hospital setting demonstrate that excessive fluid resuscitation improves outcomes (15, 17), however, there are also studies that show the opposite result (16, 37). However, in most cases the trials are inconclusive and do not support any type of treatment.

Our study refers not only to pre-hospital or perioperative periods, but also to the entire time from trauma to the end of the second day of stay in the ICU. We analyzed data until the end of the second day of stay in the ICU, since this is the time of resuscitation, stabilization, and optimization phase according to the ROSE concept of fluid therapy. On day 3 some patients already reach the de-escalation phase, which requires different fluid approaches (38). In ICU patients, positive fluid balance was associated with worse outcomes and increased mortality in many clinical trials (2, 39–42). The fluid bolus of 30 ml/kg is recommended in guidelines for the treatment of septic shock (43); however, recently this has been questioned and some publications report that such bolus may not be beneficial (44). Especially in cases of SIRS, very common in patients with serious trauma, fluids do not stay in blood vessels for long, but leak into the interstitial space, and this refers to both to colloids and crystalloids (45). Furthermore, the atrial natriuretic peptide (ANP) is secreted during hypervolemia and may be responsible for further accumulation (46).

In our study, we observed that patients with higher cumulative fluid volume or more positive fluid balance had lower number of days without mechanical ventilation and patients with higher fluid balance had additionally higher total number of complications and longer time until ready for discharge from ICU. This fact was already noticed in other studies in ICU setting (1), but not in trauma patients. Furthermore, we observed that fluid volume and fluid balance correlated positively with volume of transfused blood products, APTT, and INR values. This could be an indirect proof that massive bleeding was associated with administration of additional crystalloid or colloids, which should be avoided as it may lead to more pronounced trauma induced coagulopathy and further bleeding. This is in agreement with other studies, where fluid excess also led to coagulopathy and increased bleeding (47). We did not find a correlation between fluid volume or fluid balance and time of CRRT, which could be expected, since it has previously been shown in many studies that early initiation and longer duration of CRRT in fluid-overloaded patients with acute kidney injury has an advantageous effect and even decreases mortality (48–50). The lack of such correlation in our study might be explained by the fact that non-survivors (with higher positive fluid balance), had the CRRT stopped prematurely, before full de-resuscitation, due to death. It should be noted that the ISS in our study is relatively high compared to other studies (51, 52); however, this fact can be explained by the specification of the Level 3 Trauma Center in our hospital. The Trauma Center in our hospital is dedicated to a large region of southern Poland, where the most severe trauma victims are transported (mainly by the air transport—helicopters) in situations where very specialist treatment is necessary and when other, less specialist trauma centers in the region cannot handle them. Admission criteria are multiregional trauma and at least two of the regions must be severely injured. In addition, the evaluation of ISS is quite subjective and there could have been a trend toward overestimation in patients admitted to our Center.

Our study has many limitations, the most important of which are a retrospective design and a relatively small number of patients; therefore, some correlations could not have been detected and the results might be subject to bias. Although we included possible confounders in our analysis, we can only be sure of an association between worse outcomes and higher volumes of fluids infused; however, we cannot be sure whether excessive fluid administration actually deteriorated the treatment results. Additionally, we did not address the issue of the type of fluid used and, yet, differences may exist between colloids and crystalloids.

## 6. Conclusion

In our study, we demonstrated that in critically ill trauma patients, worse outcomes were associated with higher volumes of infusion fluids and more positive fluid balance. Even though fluid resuscitation is lifesaving, especially in the first hours after trauma, fluid infusion should be limited to a necessary minimum to avoid fluid overload and its negative consequences. Our findings emphasize the importance of avoiding unnecessary fluid overload in critically ill trauma patients; however, larger, prospective, randomized clinical trials are needed to address this problem. A well-structured and individualized approach is needed to guide physicians in fluid therapy in this clinically challenging situation.

## Data availability statement

The raw data supporting the conclusions of this article will be made available by the authors, without undue reservation.

## Ethics statement

The studies involving human participants were reviewed and approved by the Jagiellonian University Ethics Committee. Written informed consent for participation was not required for this study in accordance with the national legislation and the institutional requirements.

## Author contributions

AW, JG, TD, WS, and JD collected the patient data and imputed it into the database. AW, JG, TD, MP, JWoro, JWord, and RZ analyzed and interpreted the patient data. MP, TD, and AW performed the statistical analyzes. AW, TD, JG, JWoro, JWord, and RZ were the main contributors. All authors read and approved the final manuscript.
